# Targeting the microbiome in pediatric migraine: gastrointestinal manifestations and the therapeutic role of ***Bifidobacterium longum***

**DOI:** 10.1080/19490976.2025.2606487

**Published:** 2025-12-27

**Authors:** Pi-Chuan Fan, Huey-Huey Chua, Chia-Ray Lin, Tzu-Hsuan Lai, Lih-Chu Chiou, Wang-Tso Lee, Huey-Ling Chen, Yen-Hsuan Ni

**Affiliations:** a Division of Neurology, Departments of Pediatrics, National Taiwan University Hospital, Taipei, Taiwan; b Departments of Pediatrics, College of Medicine, National Taiwan University, Taipei, Taiwan; c Division of Gastroenterology, Departments of Pediatrics, National Taiwan University Hospital, Taipei, Taiwan; d Department and Graduate Institute of Pharmacology, College of Medicine, National Taiwan University, Taiwan; e Graduate Institute of Brain and Mind Sciences, College of Medicine, National Taiwan University, Taipei, Taiwan; f Graduate Institute of Acupuncture Sciences, China Medical University, Taichung, Taiwan; g Graduate Institute of Medical Education and Bioethics, College of Medicine, National Taiwan University, Taipei, Taiwan

**Keywords:** Gut microbiota, gut–brain axis, *Faecalibacterium prausnitzii*, *Streptococcus gallolyticus*, pediatric migraine, gastrointestinal disorder

## Abstract

Migraine is a disabling neurological disorder that often begins in childhood or adolescence and is frequently accompanied by gastrointestinal (GI) symptoms. However, the microbiota signatures and gut–brain interactions underlying pediatric migraine, particularly in the presence of GI disorder, remain poorly defined. This study aimed to explore the clinical and microbial features of pediatric migraine, as well as the therapeutic potential of probiotics.

We prospectively enrolled 126 pediatric migraine patients (ages 6–19) with or without GI disorder and 50 age-matched healthy controls. Fecal microbiota was profiled using 16S rRNA sequencing. Patients with migraine were stratified based on Rome IV-defined GI disorders and evaluated for headache characteristics, PedMIDAS scores (disability assessment), plasma calcitonin gene related peptide (CGRP, thought as a key biomarker of migraine), cytokines, and fecal calprotectin. Probiotic effects were tested in both young (3–4 weeks) and adult capsaicin-induced migraine rat models, and an exploratory pilot study involving 23 pediatric migraine patients.

Compared to controls, migraine patients exhibited distinct gut microbiota with reduced *Bifidobacterium longum.* and elevated *Bacteroides*. GI disorders were present in 46.8% of migraine patients and were associated with significantly higher rates of abdominal pain (50% vs. 13%, *p* <0.001), greater migraine-related disability (PedMIDAS: 60 ± 13.2 vs. 29 ± 7.0, *p* = 0.042), elevated fecal calprotectin, and enrichment of *Streptococcus gallolyticus*. In contrast, *Faecalibacterium prausnitzii*, positively correlated with *B. longum*, was linked to milder symptoms and shorter disease duration in migraine patients without GI disorder. In animal models, *B. longum* attenuated trigeminal activation in both young and adult rats. An exploratory pilot study showed *B. longum* supplementation led to reductions in headache days, intensity, and frequency. These findings reveal distinct gut microbial signatures in pediatric migraine, and identify *B. longum* as a promising microbiota-targeted therapeutic strategy. Our work highlights the therapeutic potential of modifying the gut–brain axis in childhood migraine.

## Graphical abstract

**Figure:**
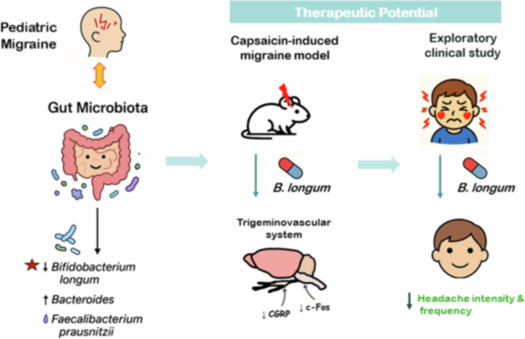

## Introduction

Migraine is a complex neurologic disorder characterized by recurrent, often disabling headaches accompanied by heightened sensitivity to light, sound, and motion.^1^ It remains among the most prevalent causes of disability worldwide.^2^ Current models suggest that migraine arises through a cascade involving peripheral trigeminovascular activation including trigeminal ganglia (TG), neurogenic inflammation, and subsequent sensitization of central pain pathways such as trigeminocervical complex (TCC). Calcitonin gene-related peptide (CGRP), a key neuropeptide in this process, contributes to vasodilation and inflammatory signaling.^3^^,^^4^ Elevated CGRP levels have been documented in both plasma and cerebrospinal fluid of migraine patients,^5-7^ particularly during acute attacks.^8^^,^^9^

A growing body of evidence indicates a strong association between migraine and gastrointestinal (GI) disorders, including functional dyspepsia, irritable bowel syndrome, and abdominal migraine,^10^ and even early-life conditions such as infant colic.^11^ These overlaps suggest shared pathophysiological mechanisms centered on visceral hypersensitivity and neuroimmune interactions, with contributors such as serotonin, CGRP, and inflammatory cytokines.^12^ The gut microbiota has emerged as a key player in brain–gut communication, influencing immune function, intestinal permeability, and neuroactive metabolite production.^13^ Increased intestinal permeability and inflammatory responses, probably mediated by proinflammatory cytokines such as tumor necrosis factor (TNF)-*α*, undigested food particles, and bacterial metabolites or endotoxins might act on the trigeminovascular system to trigger migraine-like attacks.^14-16^ Microbial metabolites such as short-chain fatty acids, serotonin, and dopamine can further signal through the enteric and central nervous systems, thereby modulating brain function.^17^^,^^18^ Early-life microbiota development also shapes long-term immune and neurologic outcomes.^19^ Despite these connections,^10^ the role of the gut microbiome in pediatric migraine, particularly in relation to GI disorders, remains poorly understood and has not been systematically characterized.

Preclinical migraine models, including nitroglycerin (NTG)-induced hyperalgesia, cortical spreading depression (CSD), and capsaicin-induced trigeminovascular activation, reproduce core features of the disorder such as CGRP release, neuronal sensitization, and migraine-like pain behaviors. Their responsiveness to established antimigraine therapies, supports their translational relevance.^20-22^ These models have recently been extended to investigate the gut–brain axis, where manipulation of the gut microbiota through antibiotics, probiotics, or fecal microbiota transplantation has been shown to modulate migraine-like phenotypes.^23^^,^^24^ Microbiome and metabolome profiling in NTG and related models further reveals taxa- and metabolite-level alterations such as short-chain fatty acids and tryptophan pathways. These changes mirror patient findings and underscore the models’ utility for studying links between gut dysbiosis and migraine pathophysiology.^25^

We hypothesized that gut dysbiosis contributes to migraine pathogenesis, and microbiome modulation may restore the deleterious effects. To test this, we conducted a prospective study combining clinical and microbial analyzes, along with an exploratory pilot study.

## Methods

### Study subjects

**Figure 1:**
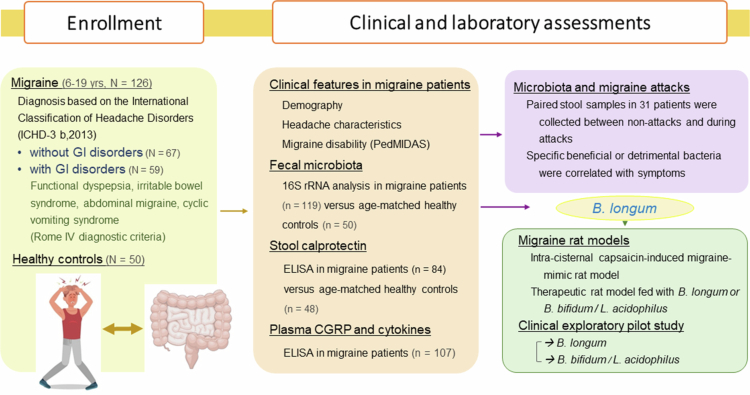
Workflow of the present study. *N*, number of patients recruited; *n*, number of stool or blood samples.

We prospectively recruited 126 patients aged 6-19 years who visited the pediatric neurology clinic or wards and diagnosed with migraine between July 2019 and October 2023 at National Taiwan University Hospital, a tertiary medical center in Taiwan. The diagnosis of migraine was made according to the criteria by the Headache Classification Committee of the International Headache, 2013.^11^ All enrolled subjects received thorough clinical evaluation and documentation, which included age, sex, age of onset, headache features with intensity scale of 0-10, duration, frequency, triggers, and family history of migraine. Migraine disability was evaluated with the Pediatric MIgraine Disability ASsessment (PedMIDAS).^26^ Fifty age-matched healthy controls were recruited with no history of headache or chronic medical, neurological, or psychiatric conditions. Both patients and controls were excluded if they had received antibiotic treatment within the previous two weeks, probiotic treatment within the past month, or if they had multiple congenital anomalies or intellectual disability (Figure 1).

To distinguish migraine patients with or without GI disorders, we recorded GI symptoms in all patients. Migraine patients with GI disorders were defined by a clinical diagnosis of migraine, according to the criteria by the Headache Classification Committee of the International Headache in 2013,^11^ plus a positive GI diagnosis including functional dyspepsia, irritable bowel syndrome, or abdominal migraine, according to Rome IV diagnostic criteria.^27^ This study was approved by the Institutional Review Board of National Taiwan University Hospital (201812147RIN), with informed consents obtained.

### Sample collection

Of the 126 patients with migraine, blood (*n* = 107) and fecal material (*n* = 119) were collected after enrollment. For microbiome analysis, fecal samples were collected from 50 age-matched pediatric healthy controls. Of the 126 enrolled patients with migraine, 31 of the subjects have had paired stool and blood samples collected, clearly linked with symptoms: between attacks and during headache attacks, with a symptom-free period of at least one week.

## Stool microbiome analysis

### 16S rRNA gene amplification and library construction

DNA was isolated from fecal samples of the migraine patients using the QIAamp PowerFecal Pro DNA kit (QIAGEN, Germany). The V3-V4 hypervariable region of the bacterial 16S ribosomal RNA gene was amplified by PCR; and the libraries for 16S V3-V4 amplicons were prepared following the Illumina library construction protocol.

### MiSeq-based high-throughput sequencing and data analysis

Libraries were sequenced using the paired-end 2 × 300 bp Illumina MiSeq platform. Raw FASTQ files were denoised using DADA2 within the QIIME 2 plugin.^28^^,^^29^ The taxonomies of denoised amplicon sequence variants (ASVs) were classified based on the EZBiocloud database.^30^ The beta diversity results were plotted with the QIIME2 plugin core-metrics-phylogenetic, and LEfSe was analyzed by using MicrobiomeAnalyst web tools.^31^

### Measurement of plasma CGRP and cytokine levels

Plasma samples from enrolled patients were stored at –80 °C and further used to measure CGRP, interleukin (IL) 1b, TNFα, prostaglandin E2 (PGE2), IL6, and NFkB levels using ELIZA kits (CGRP: Bertin bioreagent, France; IL1-b/IL6/PGE2: Abcam, UK; TNF-α: Invitrogen, U.S.A.; NFkB: Elabscience, U.S.A.). Fecal samples were also measured by ELIZA (Bühlmann, Switzerland) for calprotectin. The individual who conducted the measurement was blinded to the identity, attack status and treatment of the study subjects.

### Intra-cisternal capsaicin induced migraine-mimic rat model

All animal care and experimental protocols were approved by the Institutional Animal Care and Use Committee of the College of Medicine of National Taiwan University. Male Wistar rats aged 7-8 weeks (226−275 g) and 3-4 weeks (76−125 g) were used. Female rats were excluded as sex hormones are significant confounding factors to migraine. Group allocation was concealed from all personnel except the allocator. Blinding was maintained during the experiment, outcome assessment, and data analysis to minimize bias. Adjusted Formula (for repeated measures design), 3 measurements per animal, and an intra-animal correlation of 0.5, the required sample size is approximately 5 animals per group to estimate a sample size for an independent groups design (e.g., treatment vs. control) for our animal study. Rats were housed in a temperature-controlled facility on a 12-h light/dark cycle, with free access to standard chow (LabDiet 5010), water, and a wooden stick for gnawing. Three animals were housed per cage (47.5 × 25.8 × 21.2 cm), and cages were cleaned according to standardized procedures.

Rats were pretreated with the probiotic, *B. longum* (3 × 10^9^ colony-forming units (Tadas^TM^, Suntory, Japan), or a multispecies probiotic product containing *Bifidobacterium bifidum* (10^9^ CFU) and *Lactobacillus acidophilus* (10^9^ CFU) (abbreviated as *BbLa*, Infloran^TM^, Laboratorio Farmaceutico, Italy) as a *B. longum-*negative probiotics control by oral gauge intragastric intubation for two weeks. Half doses of probiotics were adjusted for the young group. In the treatment-naïve group, the rat was pretreated daily with water by oral gavage for two weeks. Trigeminovascular activation in rats was induced by capsaicin via intracisternal instillation, following established protocols.^22^^,^^32^^,^^33^ Rats were anesthetized with urethane (1.5 g/kg, *i.p.*) and catheterized (PE-10, SIMS Portex Ltd, Hythe, UK), with capsaicin (10 nmol, 100 μl) administered into the cisterna magna over 1 min. Distribution was facilitated by positioning the rat at –30 degrees for 30 minutes, followed by 90 minutes in a prone position. Sham rats received vehicle only. Two-hour post-instillation, rats were euthanized and perfused with paraformaldehyde (4%) via the ascending aorta for immunostaining. Outcome measures included c-Fos immunoreactive (ir) neurons in TCC, and CGRP-ir neurons in TG, all clearly defined and standardized for consistency.

### Immunohistochemical analysis of c-Fos protein in TCC tissue sections

TCC sections preparation and c-Fos-specific immunohistochemistry were accomplished following established methods.^22^^,^^32^^,^^33^ Brainstem with attached cervical cord was dissected into serial sections (50 μm) using a cryostat (LEICA CM3050S, Nussloch, Germany) from 1 mm rostral to the obex and continued to the C6 segment of the spinal cord. Sections at + 0.6, –1.2, and –9 mm from the obex were immunostained with antibody against c-Fos. The total number of c-Fos immunoreactive TCC neurons was estimated using a previously derived formula: 16(N1 + N2)/2 + 53(N2 + N3)/2, where N1, N2, and N3 were c-Fos-ir neuronal numbers at + 0.6, –1.2, and –9 mm from the obex, respectively.^22^

Free-floating immunohistochemistry for c-Fos protein was performed using the avidin-biotin method following the established protocols.^22^ Detection was performed using a rabbit polyclonal anti-c-Fos antibody (1:500 dilution, Abcam, Cambridge, UK, RRID: AB_306177), biotinylated anti-rabbit IgG (1:200, Abcam, Cambridge, UK, RRID: AB_2313606), and horseradish peroxidase avidin D (1:500, Abcam, Cambridge, UK, RRID: AB_2336507). Immunoreactive signals were visualized with the DAB Reagent kit (KPL, Gaithersburg, MD, U.S.A.), and c-Fos-ir neurons were counted using a microscope (Olympus BX51, Essex, UK). Data analysis was conducted by an investigator blinded to the treatment groups.

### Immunofluorescence of CGRP in TG slice sections

TG sections preparation and CGRP immunofluorescence quantification followed established methods.^22^^,^^32^^,^^33^ Two TG tissues were dissected per rat and sectioned at a thickness of 50 μm using a microtome (LEICA RM2245, Nussloch, Germany). Nine sections were sampled from each ganglion, with every third section used for CGRP immunofluorescence. The sections were incubated with a rabbit primary antibody against CGRP (1:200; EMD Millipore, Burlington, MA, U.S.A.) and then with a fluorescein-conjugated goat anti-rabbit IgG secondary antibody (1:50; Vector Labs, Burlingame, CA, U.S.A.). Images were captured using an inverted light microscope (Zeiss Axio Observer. D1; Carl Zeiss, Jena, Germany), and the intensity of CGRP expression was analyzed at 100X magnification using ImageJ software. The analysis was based on the optical density multiplied by the activated areas in each image.

### Drugs

Capsaicin purchased from Sigma Chemical (St. Louis, MO, U.S.A.) was dissolved in a vehicle containing 10% ethanol and 10% Tween 80, sonicated for 5 min, and then further diluted (1:100) in an artificial CSF (aCSF) to create a stock solution which was stored at 4 °C. The aCSF comprised the following components (in mm): 117 NaCl, 4.5 KCl, 2.5 CaCl_2_, 1.2 MgCl_2_, 1.2 NaH_2_PO_4_, 25 NaHCO_3_, and 11.4 dextrose bubbled with 95% O_2_/5% CO_2_, pH 7.4.

### 16S rRNA microbiome analysis in mouse stools

Fecal samples were collected into sterilized 1.7 mL copolymer microtubes before anesthesia for surgical procedures and stored at −80 °C within 4 hours of collection. DNA was extracted using the QIAamp PowerFecal Pro DNA Kit (QIAGEN). DNA concentration was quantified with a Qubit fluorometer, and libraries were prepared for 16S rRNA V3–V4 sequencing using the same protocol as described for migraine patient samples.

### An exploratory pilot study to assess the clinical benefit of *B. longum*

The effect of *B. longum* was evaluated in an exploratory randomized double-blind pilot study assessing the feasibility of using probiotics in migraine patients who met the ICHD-3 criteria,^11^ between January 2023 and February 2025. This study was approved by the Research Ethics Committee of National Taiwan University Hospital (202207086RIND) (NCT07092241). Signed informed consent was obtained from each patient and the parents of the pediatric patients. Participants were randomly assigned (3:1) to take *B. longum* (Tadas®, Suntory) or a formulation containing *B. bifidum* and *L. acidophilus* (Infloran®, Laboratorio Famaceuticr), the latter as a control group, using a computer-generated sequence. Allocation was concealed using sequentially numbered, sealed envelopes. Participants received daily intake of one of two probiotic products for 12 weeks: The probiotics in the latter formulation (*B. bifidum* and *L. acidophilus*) were not associated with migraine benefit in either previous or current studies. Throughout the study period, patients maintained a daily headache diary documenting pain score (0–10 scale), headache frequency, and number of headache days per week.

### Statistical analysis

Data assumptions were assessed using normality tests (e.g., Shapiro-Wilk, or Kolmogorov-Smirnov) for continuous variables and homogeneity of variance (e.g., Levene’s test). If assumptions were violated, non-parametric tests (e.g., Mann-Whitney U test or Wilcoxon signed-rank test) were applied as appropriate. Data are expressed as the means ± SEs unless otherwise specified. All categorical variables were analyzed with chi-square tests or Fisher's exact test. Results with a *p* value <0.05 was considered statistically significant. The statistical analyzes were performed using SPSS (version 20.0; SPSS Inc., Chicago, IL). Linear discriminant analysis (LDA) effect size (LEfSe) was used to identify specific microbes that best discriminated the groups. LDA score > 4 was defined as significant abundance. A linear mixed-effects model was fitted using the lme4 package (version 1.1−37) in R to estimate least squares means (LS means). The LS means were estimated using the emmeans package (version 1.11.1).

## Results

### Distinctive microbiota features involved in migraine pathogenesis

#### Study population

To explore the impact of gut microbiota on the pathogenesis of migraine, we enrolled 126 patients with migraine and 50 age-matched healthy controls in this study (Figure 1). Migraine patients were classified into subgroups based on the presence (*n* = 59) or absence (*n* = 67) of GI disorder according to their clinical manifestation (Figure 1). Patients were followed up for at least 6 months, and fecal samples were collected during both the attack (A) and non-attack (NA) phases throughout the study period (Figure 1).

#### Identification of migraine-associated taxa

**Figure 2.**
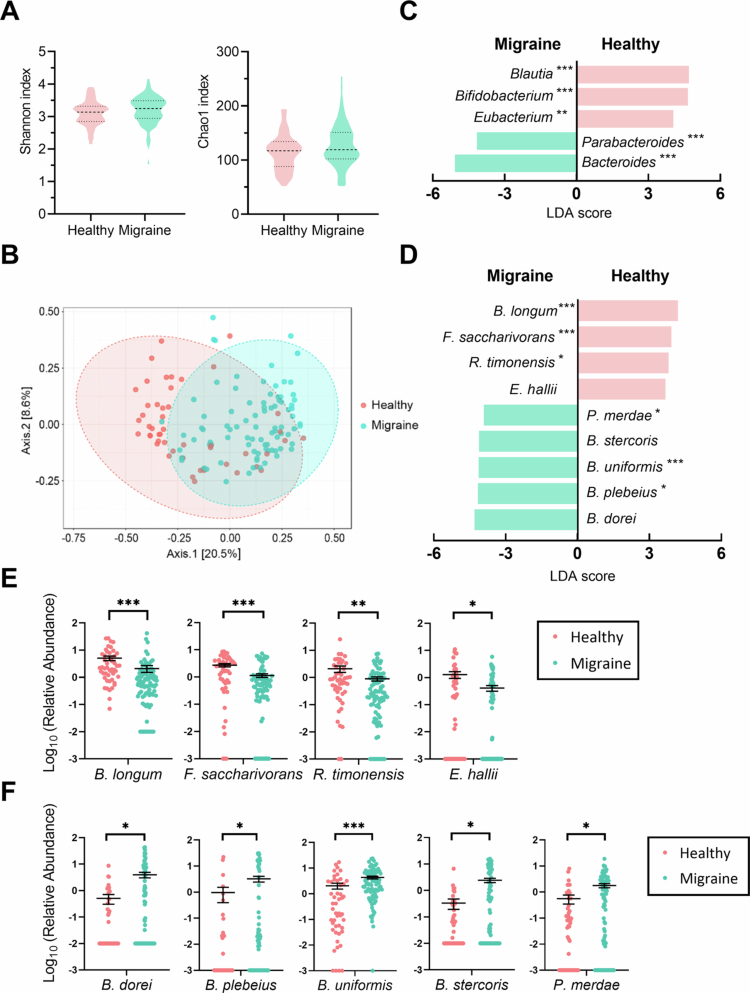
Richness and diversities of gut microbiota in migraine patients and healthy controls. (A) Shannon and chao1 indexes accessed microbial richness at the amplicon sequence variant (ASV) level. (B) Principal coordinates analysis (PCoA) using bray-cutis distance matrix distinguishes microbial diversities between groups. (C, D) Linear discriminant analysis (LDA) effect size (LEfSe) identified discriminating taxa at genus (C) and species (D) levels (*p* <0.05 and LDA > 4.5 considered significant). *B. longum*, *Bifidobacterium longum*; *F. saccharivorans, Fusicatenibacter saccharivorans*; *R. timonensis*, *Romboutsia timonensis*; *E. hallii, Eubacterium hallii*; *P. merdae, Parabacteroides merdae; B. stercoris, Bacteroides stercoris; B. uniformis, Bacteroides uniformis; B. plebeius, Bacteroides plebeius; B. dorei, Bacteroides dorei.* Statistical significance using FDR correction: * *q* <0.05; ** *q* <0.01; *** *q* <0.001. (E, F) Relative abundances (log_10_ ± SEM) of taxa enriched in healthy controls (E) or migraine patients (F). *p <0.05, **p <0.005, ***p <0.0005 (Mann-Whitney U-test).

In the microbiota analysis, we observed no significant difference in alpha-diversity between the migraine and healthy control groups, as indicated by the Shannon and Chao1 indexes at the amplicon sequence variant (ASV) level (Figure 2A), suggesting similar richness between the two groups. Of note, PCoA based on Bray–Curtis distance matrix, which identified beta diversity differences, revealed a distinct separation of the migraine samples from those of the healthy controls (Figure 2B), highlighting the variability in bacterial composition under migraine stress.

To identify migraine-related taxa, LEfSe was employed to generate a cluster diagram illustrating the differential bacterial genera and species between the groups (Figure 2C,D). Genera such as *Blautia, Bifidobacterium*, and *Eubacterium* were found in abundance among healthy controls, while *Parabacteroides* and *Bacteroides* were enriched in migraine patients (Figure 2C).

Upon further species identification, LEfSe revealed that *Bifidobacterium longum* and *Fusicatenibacter saccharivorans* were the predominant bacteria distinguishing healthy controls from migraine sufferers (Figure 2D). Besides, *Bacteroides dorei*, *Bacteroides plebeius*, *Bacteroides uniformis*, *Bacteroides stercoris*, and *Parabacteroides merdae* a were overrepresented in patients with migraine (Figure 2D). Dot plots further illustrated these remarkable differences (Figure 2E,F).

The colonization of *B. longum*, which was significantly higher in healthy controls than the migraine patients (Figure 2E), suggesting a potential for supplementation of *B. longum* to reconstruct the dysbiotic gut microbiota of patients with migraines. Although the level of *F. saccharivorans* in healthy controls was more abundant than that of migraine patients (Figure 2E), its probiotic role was still poorly understood.

### *B. longum* ameliorates pain induced by capsaicin in migraine rat model

#### Capsaicin-induced migraine rat model

**Figure 3.**
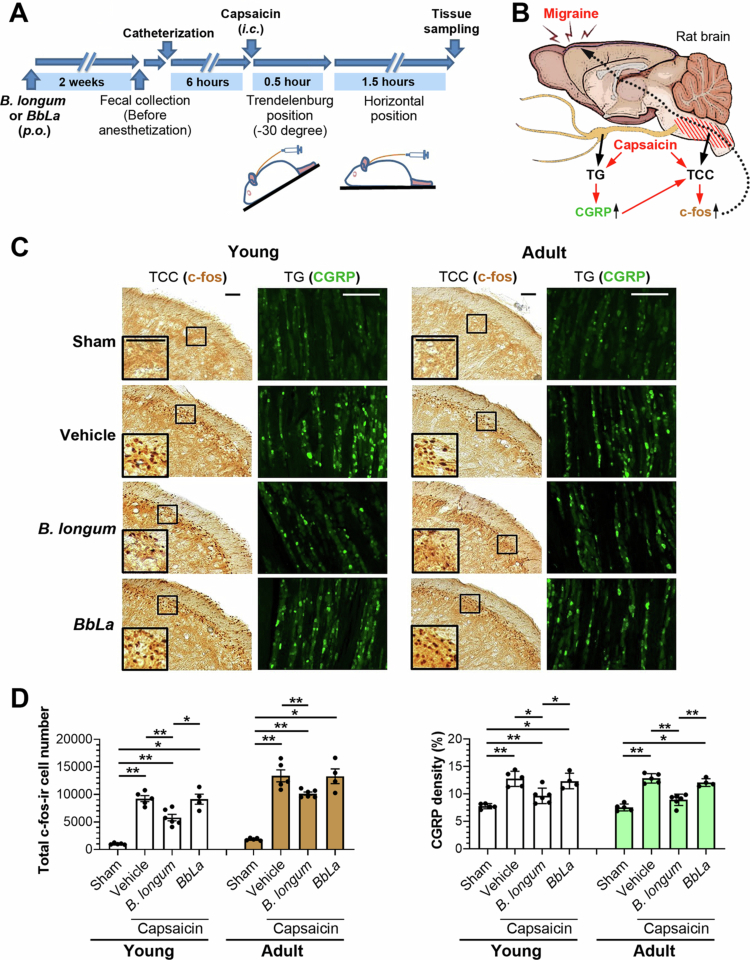
Effect of *B. longum* on capsaicin-induced neuronal activation in the trigemino-cervical complex (TCC) and trigeminal ganglia (TG). (A) Experimental design: male Wistar rats (adult 7-8 wk, young 3-4 wk) were pretreated for 2 wk with *B. longum* (3 × 10^9^ CFU adults; 1.5 × 10^9^ CFU young) or *BbLa* (250 mg containing *B*. *bifidum* (10^9^ CFU) and *Lactobacillus acidophilus* (10^9^ CFU) adults; 125 mg young) via oral gauge. Rats (*n* = 4-6/group) then received intracisternal capsaicin (10 nmol). (B) Schematic of migraine pathology showing increased c-Fos and CGRP in the trigeminal system. (C) c-Fos immunohistochemistry (TCC) and CGRP immunofluorescence (TG). Sham rats received vehicle instead of capsaicin. Three TCC and TG sections was analyzed per rat. Scale bar: 100 µm. (D) Quantification of c-Fos-immunoreactive TCC neurons and CGRP density in TG sections. CGRP density (%) = (activated cell area/full cell area) × 100% in V1 + V2 region. Data are means ± SE. *p <0.05, **p <0.005 (Mann-Whitney U-test).

In order to test whether *B. longum* administration improves the migraine headaches, a relevant rat model for migraine was established. Male Wistar rats (adults aged 7 to 8 weeks and young rats aged 3 to 4 weeks, *n* = 4-6/group, 2-3 rats per cage) were orally gavaged daily with *B. longum* or a probiotic control containing *BbLa* for 2 weeks prior to intracisternal administration of capsaicin (Figure 3A). This capsaicin-induced migraine-mimicking rat model had been well-characterized by increasing the immunoreactivity of c-fos in TCC neurons^22^^,^^33^^,^^34^ and CGRP in TG,^22^^,^^33^ as illustrated in the schematic in Figure 3B.

#### Anti-migraine effect of B. longum

It is noteworthy that *B. longum* feeding significantly reduced the immunoreactive (ir) neurons of c-fos and CGRP in TCC and TG, respectively (Figure 3C,D). This finding was reproducible in both young and adult rat models (Figure 3C,D), suggesting an anti-migraine effect of *B. longum*.

#### B. longum treatment changes the gut microbiota composition

**Figure 4.**
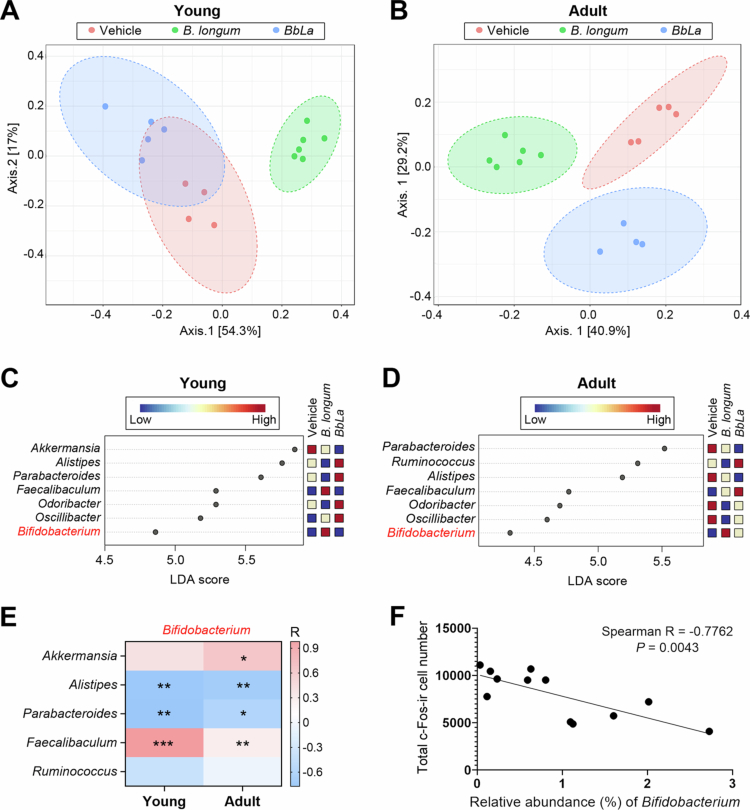
*B. longum* alters gut microbiota composition and diversity in a migraine rat model. (A, B) PCoA plots based on bray-curtis distance matrix showing beta diversity of fecal microbiota in young (A) and adult (B) rats. (C, D) Genus-level taxa with LDA thresholds of 4.5 (young, C) and 4 (adult, D). (E) Heatmap of Spearman rank correlations coefficient *r* between *Bifidobacterium* and other microbiota genera detected in the fecal samples of young and adult rats. (F) Linear regression depicts the negative correlation between *Bifidobacterium* abundance and total c-Fos-ir cell number in TCC sections of young and adult rats that fed with *B. longum.* *p <0.01, **p <0.001, ***p <0.0001 (Mann-Whitney U-test).

It is important to pursue how *B. longum* blocked the capsaicin-activated migraine pain. We assumed that the reconstruction of gut microbiota induced by *B. longum* could prevent or alleviate the migraine pain. Accordingly, we analyzed the gut microbiota constitution of the aforementioned migraine rat models. Their fecal samples were collected before anesthesia for surgical procedures and the gut microbiota were revealed by 16S rRNA NGS profiling. PCoA analysis based on the Bray–Curtis method demonstrated significant clustering among vehicle-, *B. longum*- and BbLa-fed groups in both young and adult rats (Figure 4A,B).

#### Differentially abundant genera in response to B. longum

A threshold of at least 4 on the logarithmic LDA score was adopted for discriminative features. At the genus level, *Akkermansia* and *Parabacteroides* had the highest LDA score for the young and adult migraine rats, respectively (Figure 4C,D). Feeding of *B. longum* gave rise to an elevation of *Bifidobacterium* level in both young and adult rats as compared to those of vehicle treatment (Figure 4C,D).

Of note, the abundance of *Bifidobacterium* in both young and adult rats was negatively correlated to the *Parabacteroides* (Figure 4E), which was highly colonized in the migraine patients (Figure 2C), hinting that *Bifidobacterium* may impede the growth of migraine-associated *Parabacteroides* and improve the microbial balance in gut of the host. Importantly, the abundance of *Bifidobacterium* was positively correlated with the *Faecalibaculum* level in both young and adult rats (Figure 4E).

#### Correlation with neuronal activity

In addition, the fecal levels of *Bifidobacterium* of both young and adult rats were significantly inversely associated with the number of c-fos-ir neurons in the TCC (Spearman *r* = -0.7762, *p* = 0.0043, Figure 4F), further emphasizing the potential anti-migraine role of *B. longum*.

### Gut microbiota differences between migraine patients with and without GI disorders

#### Clinical characteristics of migraine patients with and without GI disorders

Among the 126 migraine patients, we found that 59 patients (46.8%) were designated as the group with GI disorders (M + G), whereas 67 (53.2%) were without GI disorders (M + noG). There were no differences in the age, sex, onset durations, pain scores, and medication used between M + G versus M + noG groups. (Table 1) Medication used included anti-migraine, vitamins, and anti-allergic drugs. Abdominal pain significantly differentiated migraine patients with GI disorders from those without (50% vs. 13%, *p* <0.001) (Table 1), while nausea/vomiting were prevalent in both groups (57% vs. 59%, *p* = 0.861). Patients with GI disorders had higher PedMIDAS scores (60 ± 13.2 vs. 29 ± 7.0, *p* = 0.042).

**Table 1.** Demographics and clinical characteristics of the enrolled migraine patients with and without GI disorders.

All *N* (%)	Total migraineurs (*N* = 126)	Migraine with GI disorder (*N* = 59)	Migraine without GI disorder (*N* = 67)	*P* value^a^
**Sex (Male/Female)**	61/65	25/34	36/31	0.203
**Age (months)**	143 ± 3.4	145 ± 4.8	141 ± 4.8	0.562
**Age of onset (m)**	112 ± 3.7	110 ± 5.3	114 ± 5.2	0.622
**Onset duration (m)**	30.9 ± 2.6	34.6 ± 4.6	27.7 ± 2.9	0.208
**Attacks over 3 m**	28.8 ± 2.6	28.6 ± 3.6	28.9 ± 3.8	0.95
**Pain score (0−10)**	5.87 ± 0.16	6.14 ± 0.23	5.64 ± 0.23	0.124
**PedMIDAS** ^b^	43 ± 7.3	60 ± 13.2	29 ± 7.0	0.042*
**Medication use**	80 (65)	37 (65)	43 (64)	0.932
**Migraine characters**				
**Duration: <4 h: 4hr-1d: >1 d**	64:32:27	27:19:11	37:13:16	0.226
** Aura**	45 (36)	25 (44)	20 (30)	0.134
** Location**				
** frontal**	52 (44)	20 (36)	32 (51)	0.115
** temporal**	74 (63)	36 (67)	38 (60)	0.478
** parietal**	40 (34)	14 (24)	26 (41)	0.07
** occipital**	49 (41)	25 (46)	24 (38)	0.379
** Unilateral**	17 (16)	10 (19)	7 (13)	0.360
** Throbbing**	58 (48)	29 (53)	29 (45)	0.376
** Activity-aggravated**	89 (72)	43 (75)	46 (69)	0.675
** Nausea/vomiting**	67 (58)	31 (57)	36 (59)	0.861
** Abdominal pain**	35 (30)	27 (50)	8 (13)	<0.001*
** Photophobia**	39 (34)	20 (37)	19 (31)	0.506
** Phonophobia**	61 (53)	32 (59)	29 (48)	0.209
** Visual symptoms**	26 (23)	11 (20)	15 (25)	0.589
** Chest pain**	18 (16)	12 (22)	6 (10)	0.068
** Tinnitus**	26 (23)	10 (19)	16 (26)	0.324
** Vertigo**	34 (30)	15 (28)	19 (31)	0.693
** Dizziness**	71 (62)	32 (59)	39 (64)	0.607
** Weakness**	29 (25)	15 (28)	14 (23)	0.552
** Released by sleep**	90 (73)	42 (74)	48 (72)	0.938
**Food related**	20 (17)	10 (18)	10 (16)	0.803
**Allergic history**	74 (61)	32 (57)	42 (64)	0.464
**Stooling habit: daily**	82 (66)	35 (61)	47 (70)	0.305
**FHx** ^c^ **of migraine**	82 (66)	41 (72)	41 (61)	0.208
** Dad: Mom: both**	25:41:16	13:21:7	12:20:9	0.855

a Differences between migraine with and without gastrointestinal (GI) disorder groups.

b Pediatric Migraine Disability Assessment.

c Family history; *P <0.05.

#### Distinct bacterial taxa associated with GI comorbidity

**Figure 5.**
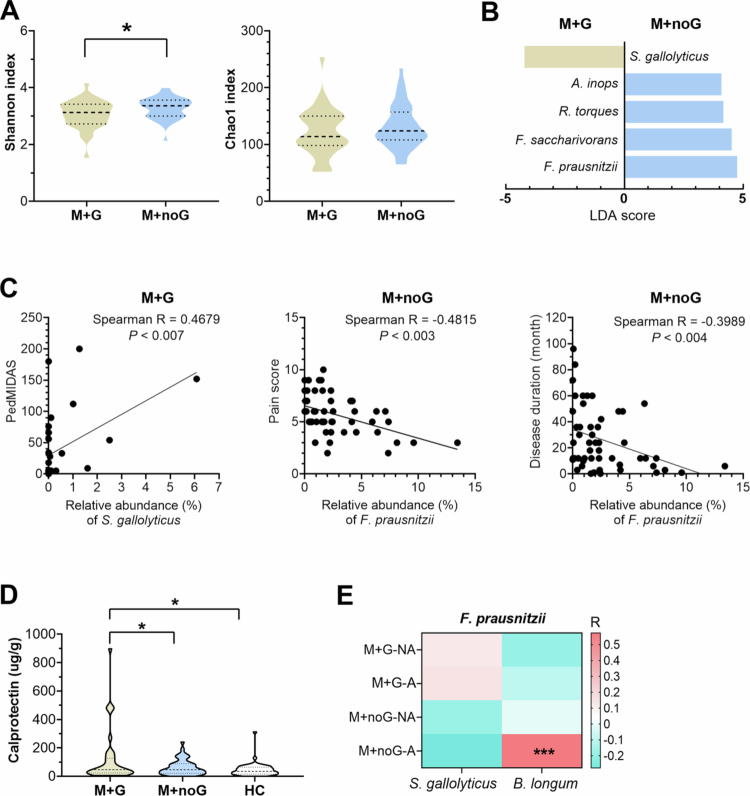
Bacterial diversity and clinical correlations between migraine patients with and without GI disorders. (A) Shannon and Chao1 indexes at the ASV level. (B) LEfSe identified discriminating taxa (*p* <0.05 and LDA > 4): *S. gallolyticus*, *Streptococcus gallolyticus*; *A. inops, Alistipes inops*; *R. torques*, *Ruminococcus torques*; *F. saccharivorans*, *Fusicatenibacter saccharivorans*; *F. prausnitzii, Faecalibacterium prausnitzii.* (C) Spearman correlations between bacteria and clinical factors (pain score, PedMIDAS and disease duration) in patients with (M + G) or without (M + noG) GI disorder. (D) Fecal calprotectin levels among M + G (*n* = 35), M + noG (*n* = 49), and healthy controls (*n* = 48). (E) Heatmap of Spearman correlations between *F. prausnitzii* and *S. gallolyticus*/*B. longum* during non-attack (NA) and attack (A) periods. *p <0.05, **p <0.005, ***p <0.0005 (Mann-Whitney U-test).

We further compared the gut microbiota profile between patients with M + G and M + noG. Shannon (*p* <0.03) and Chao1 indexes showed an increased richness of gut microbiota in fecal samples of patients with M + noG (Figure 5A).

In the LEfSe analysis, *Streptococcus gallolyticus* appeared enriched in patients with M + G, whereas patients with M + noG tended to show higher levels of *Faecalibacterium prausnitzii*, *Fusicatenibacter saccharivorans*, and others (Figure 5B). Although these findings did not reach significance after FDR correction, we noted several associations of potential biological relevance. In particular, the presence of *S. gallolyticus* in M + G patients correlated with higher PedMIDAS scores (Spearman r = 0.4679, *p* <0.007, Figure 5C), suggesting a possible link between this bacterium and more severe clinical manifestations.

Although gut microbiota profiles differed significantly between M + G and M + noG patients, plasma levels of migraine markers (CGRP and PGE2) and inflammatory indices (IL-1β, IL-6, NF-κB, TNF-*α*, supplementary Figure 1) did not. This underscores the role of gut microbiota in distinguishing the two patient groups.

#### Protective role of Faecalibacterium prausnitzii

In patients of the M + noG group, *F. prausnitzii*, was significantly linked to a reduced pain score (Spearman *r* = -0.4814, *p* <0.0003) and shortened disease duration (Spearman *r* = -0.3989, *p* <0.004) (Figure 5C). Patients with M + noG, who exhibited a higher level of *F. prausnitzii,* showed less severe intestinal inflammation. Their fecal calprotectin levels were significantly lower than those of M + G patients (*p* <0.02) and comparable to healthy controls (Figure 5D). All of these findings suggest a potential role of *F. prausnitzii* in migraine management.

Importantly, the growth of *F. prausnitzii* in M + noG patients was strongly and significantly associated with the richness of *B. longum* (Spearman *r* = 0.5731, *p* <0.0003, Figure 5E), suggesting *B. longum* may be involved in the regulation of *F. prausnitzii*. Observations from migraine rat model further support this finding. In both young and adult rats, a positive correlation between *Bifidobacterium* and *Faecalibaculum* levels was established in response to *B. longum* supplementation (Figure 4E), hinting that these bacteria may act synergistically to counter migraine attack in both humans and rats.

#### *B. longum and F. prausnitzii* associate with better clinical scores

**Figure 6.**
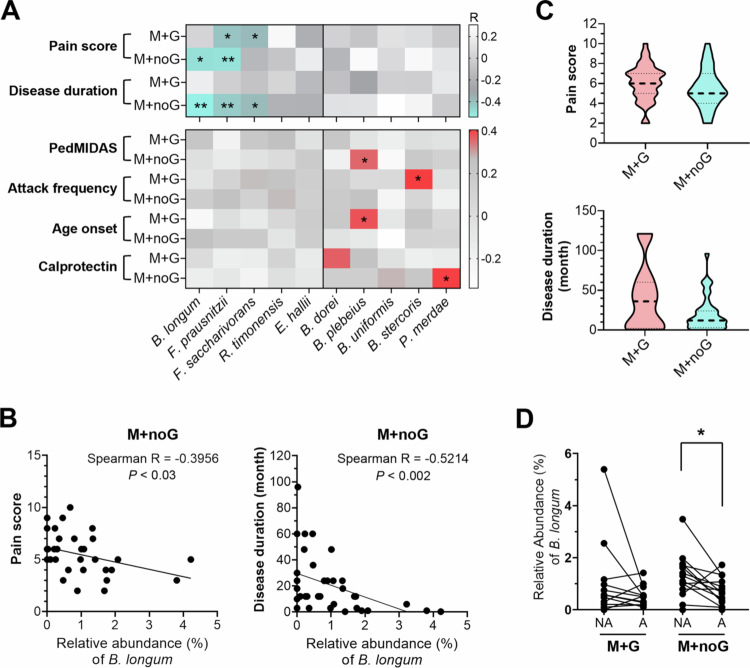
*B. longum* negatively associates with migraine activity of patients without GI disorders. (A) Heatmaps of Spearman rank correlation *r* values between gut microbiota and clinical variables indicated. Fecal samples collected during attack phase of migraine patients with GI disorders (M + G) and those without GI disorders (M + noG) were analyzed. *p <0.05, **p <0.005 by Mann-Whitney U-test. (B) Linear regression plots depicting correlations between *B. longum* and pain score (left) or disease duration (right) of M + noG patients during attack phase. (C) Violin plots show the range and distribution of pain score (upper) and disease duration (lower) of patients with M + G or M + noG. (D) A comparison of the relative abundance (%) of *B. longum* detected during non-attack (NA) and attack (A) stages of M + G and M + noG patients.

To test the aforementioned hypothesis, the importance of *B. longum* and *F. prausnitzii* in migraine patients during the attack period was specifically studied using Spearman correlation coefficient analysis. A significant negative association between *B. longum* and pain score (Spearman *r* = -0.3956, *p* <0.03), as well as disease duration (Spearman *r* = -0.5214, *p* <0.002), was observed in patients with M + noG (Figure 6A,B), similar to the associations observed for *F. prausnitzii* (Figures 5C and 6A). Additionally, *F. saccharivorans* further contributed to the negative association of the disease duration and pain score of M + noG and M + G patients (Figure 6A). In combination of the effects of *F. prausnitzii*, *B. longum* and *F. saccharivorans*, a trend of decreasing pain score and disease duration was observed in patients with M + noG as compare to those with M + G (Figure 6C). Although *F. prausnitzii* and *F. saccharivorans* contributed to the reduction in pain scores in M + G patients, they could not achieve the same level of reduction as observed in M + noG patients presumably due to the absence of *B. longum* involvement (Figure 6A and C).

In addition, the growth of *B. longum* in M + noG patients was more rapid during non-attack intervals but significantly decreased after transitioning into the attack stage (Figure 6D). This suggests the importance of maintaining a high level of *B. longum* to prevent GI manifestation and even migraine attacks.

#### Pathogenic bacterial species associated with poor prognosis

On the other hand, bacteria such as *B. dorei, B. plebeius*, *B. uniformis*, *B. stercoris*, and *P. merdae* were more abundant in migraine patients (Figure 2D) and were positively associated with worse clinical outcomes, including higher PedMIDAS scores, more frequent attacks, and elevated calprotectin level (Figure 6A). These associations suggest their potential pathogenic roles in migraine attacks.

### *B. longum* appears to reduce headache burden in pediatric migraine patients

#### Study subjects

In our exploratory pilot study, with 17 migraine patients (9 female) receiving *B. longum* (BL) and 6 (3 female) receiving *B. bifidum/L. acidophilus* (supplementary Figure 2). The two groups were comparable in sex (*p* = 0.901) and age (10.8 ± 0.7 vs. 12.1 ± 1.2, *p* = 0.29, by Mann-Whitney U test) at baseline.

#### Effect of B. longum on pain scores and headache days

**Figure 7.**
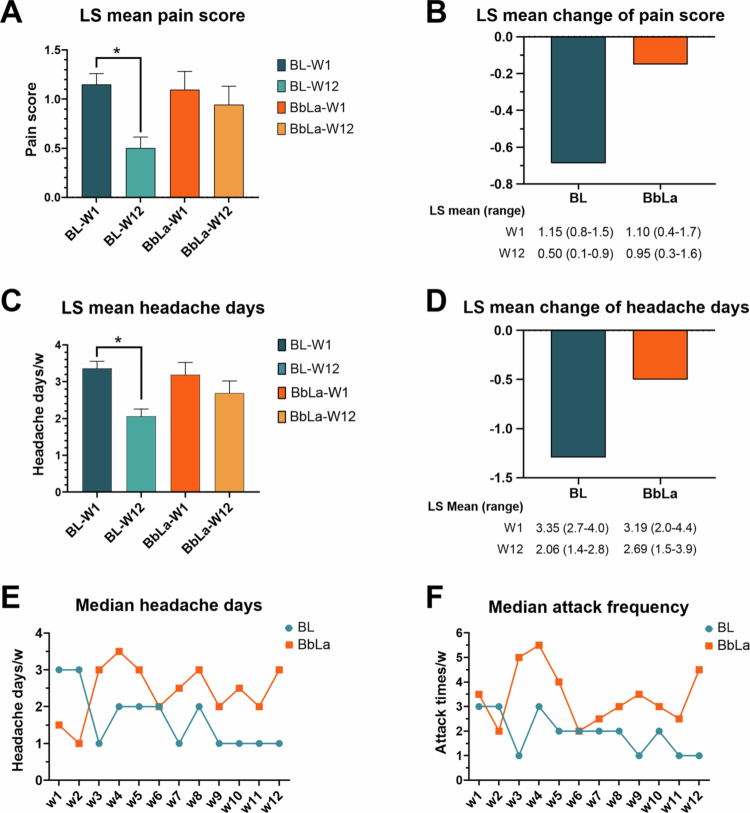
Therapeutic effects of *B. longum* in pediatric migraine patients compared to the group using *B. bifidum/L. acidophilus*. Over the 12-week study period, patients treated with *B. longum* (BL, *n* = 17) demonstrated significant improvements in clinical outcomes by least-squares (LS) mean comparison, including significant reduction of pain scores after treatment (*p* = 0.025) (A-B), and weekly headache days (*p* = 0.014) (C-D). No significant changes were observed in the group taking *B. bifidum*/*L. acidophilus* (BbLa, *n* = 6) for any parameters (all *p* > 0.05) (A-D). *p <0.05 by the Wald test. The beneficial effects of BL on weekly trends in the median number of headache days (E) and attack frequencies (F) are shown.

Patients receiving *B. longum* (7 × 10⁹ CFU) exhibited a statistically significant improvement in least-squares (LS) mean pain scores from week 1 to week 12 (Wald test, *p* = 0.025). The LS mean change was greater in the BL group (–0.64) than in the control group (–0.15). A significant reduction in the LS mean number of weekly headache days was also observed from week 1 to week 12 (Wald test, *p* = 0.014), with a greater LS mean change in the BL group (–1.29) compared to the control group (–0.5). In contrast, no significant changes were observed in the control group across any headache parameters (*p* = 0.6 and 0.75 for weekly headache days and pain scores, respectively), shown in Figure 7A−D. No serious adverse events were reported in either group.

#### Longitudinal trends in headache frequency

In addition, we found that the BL group showed a decrease after week 2, followed by stabilization at around 1 headache day per week by week 8 post-treatment (Figure 7E). In contrast, the *BbLa* group generally maintained a higher number of headache days per week, typically ranging between 2 and 3.5 days. The median weekly attack frequency showed that the BL group initially had a similar frequency to the control group but began to trend toward a lower and more stable frequency (~1–2 times/week) from Week 5 onward. Conversely, the control group exhibited greater variability and a higher frequency of attacks, with a notable peak at Week 4 (Figure 7F).

Altogether, these data suggest that the BL group experienced better outcomes in terms of reduced pain scores, fewer headache days, and lower attack frequency compared to the control group. This indicates a potential therapeutic benefit of BL treatment in managing headache-related symptoms.

## Discussion

### Novelty of the study and microbiome-related clinical features

To the best of our knowledge, this is the first study to characterize the microbiota in pediatric migraine patients with and without GI disorders. Distinct microbial and clinical profiles were identified, with *B. longum* emerging as a key species distinguishing healthy controls from migraine patients. Application of *B. longum* to the migraine-mimicking animal model confirmed its protective roles in both adult and pediatric ages. An exploratory pilot study further showed that *B. longum* is promising to reduce headache burden in pediatric migraine patients.

To characterize the differential microbiome-related clinical features in migraine patients, we found that, among patients without GI symptoms, *B. longum* abundance was associated with milder pain and shorter disease duration, and its co-occurrence with *F. prausnitzii* suggests a beneficial microbial network. In contrast, *S. gallolyticus* was enriched in those with GI manifestations and linked to greater disability, indicating a distinct microbiota signature underlying migraine-GI comorbidity. These findings support the role of gut dysbiosis in migraine pathogenesis and highlight *B. longum* as a promising microbiome-targeted therapeutic.

### Identifying *B. longum* as a signature bacterium with therapeutic potential

In our rat model, *B. longum* attenuated neuronal activation markers such as c-Fos in the TCC and reduced CGRP expression in the TG, consistent with its known anti-nociceptive and anti-inflammatory properties.^35^^,^^36^ Clinically, pediatric migraine patients receiving *B. longum* exhibited significant improvements in pain intensity, attack frequency, and headache days, while no such effects were seen in the *B. bifidum/L. acidophilus* group, reinforcing the efficacy of *B. longum*. Previous studies have shown the psychotropic and immunomodulatory benefits of *Bifidobacterium* and *Lactobacillus* species across a range of neurological and inflammatory conditions.^35-38^ Probiotics can enhance the integrity of the intestinal epithelial barrier^39^ and may be beneficial to the gut-brain axis. However, few probiotic trials have targeted migraine.^40^ Our results align with earlier reports where *B. longum*-containing multispecies probiotics improved migraine symptoms,^41^ unlike those lacking this strain.^42^ The current findings advance this evidence by isolating *B. longum*'s specific impact.

### Gut-brain axis in migraine

Mechanistically, how gut microbes influence central migraine pathways remains incompletely understood. The proximity and connectivity between the nucleus tractus solitarius (NTS) and the trigeminal nucleus caudalis suggest a gut-brain interface modulated by microbial activity.^43^^,^^44^ Repeated unpleasant GI stimuli can cause central sensitization in the NTS,^45^ which, through its connections with the TNC, may lead to neurogenic inflammation in the dura mater and headaches.^46^ A growing body of evidence supports a biologically plausible pathway by which gut microbial alterations can modulate peripheral and central nociceptive signaling relevant to migraine. Gut microbes influence host neuroimmune signaling and metabolite profiles, notably short-chain fatty acids, which in turn can alter sensory neuron excitability and neuropeptide release. CGRP is a central effector in both visceral sensory signaling and migraine pathophysiology and is therefore a plausible mediator linking dysbiosis to headache-relevant neural activation.^47^ Preclinical work shows that gut microbes affect visceral sensitivity by regulating CGRP production, and changing the microbiota can alter CGRP-dependent signaling.^48^ This helps explain why microbiota disturbances can increase CGRP and c-Fos activity in trigeminovascular circuits. Shifts in intestinal sensory neuron activity may make vagal and spinal nerves, as well as trigeminal neurons, more sensitive, leading to greater CGRP release and the c-Fos response seen in our study. Nevertheless, plasma CGRP levels and other proinflammatory cytokines were not significantly different between migraine sufferers with and without GI disorder, implying these factors may play a shared detrimental role in migraine regardless of GI symptoms. Interestingly, evidence have shown that *B. longum* can normalize behavior in anxious mice via the vagus nerve,^38^ suggesting neurotransmission possibilities.

### Potential roles of other gut microbes

*F. prausnitzii* is being investigated as a next-generation probiotic particularly for GI diseases, due to its anti-inflammatory properties and its role in modulating metabolic processes.^49^^,^^50^ In the current study, we found the positive association of abundance of *B. longum* and *F. prausnitzii* with reduced migraine severity in the non-GI disorder group. *F. prausnitzii* is known for its ability to reduce IL-12 and IFN-*γ* production while enhancing high secretion of IL-10, an anti-inflammatory cytokine with therapeutic potential.^51^^,^^52^ Several studies have reported cooperative cross-feeding interactions between *F. prausnitzii* and *Bifidobacteria*, including *B. longum* and its subspecies.^53^^,^^54^ The commonly described mechanism is that *Bifidobacteria* ferment carbohydrates to acetate (or lactate/oligosaccharide breakdown products), which *F. prausnitzii* subsequently utilizes to produce butyrate, a beneficial short-chain fatty acid that promotes the growth of *F. prausnitzii* and *Bifidobacteria*. Therefore, we hypothesize that the enrichment of *F. prausnitzii* specifically in M + noG patients may attenuate GI inflammation and concurrently promote the expansion of *B. longum*, thereby creating a mutually beneficial environment that supports the bacterial survival.

On the other hand, *S. gallolyticus*, a known oncomicrobe,^55^ was enriched in migraine patients with GI disorder in the current study. It has been implicated in promoting colon cancer development through activation of the MAPK and PI3K/Akt/mTOR pathways,^56^ both of which are also relevant to migraine pathophysiology. Activation of MAPK signaling mediates the up-regulation of CGRP in rat TG.^57^ Pharmacological inhibition of PI3K relieves the pain hypersensitivity of chronic migraine rat model and this effect can be reversed by PI3K-specific agonist.^58^ We hypothesized that *S. gallolyticus* may exacerbate migraine pain by initiating MAPK and PI3K/Akt/mTOR cascades. Although there is no direct evidence linking *S. gallolyticus* specifically to migraine, it has been detected in central nervous system (CNS) infections, such as human meningitis.^59^ In veterinary studies, *S. gallolyticus* has been reported to cause central nervous manifestations, including severe neurological manifestations in geese,^60^ and encephalitis and meningitis in poults.^61^ Our study reveals a possible role of *S. gallolyticus* in pediatric migraine, which awaits confirmation from further clinical or mechanistic studies.

### Intestinal inflammation and GI symptoms

Elevated fecal calprotectin levels in migraine patients with GI disorders further support a role for mucosal inflammation. Calprotectin, a neutrophil-derived protein^62^ and biomarker of intestinal inflammation,^63^ has also been associated with pediatric episodic syndromes like infantile colic,^64^ recognized migraine variants. Thus, intestinal inflammation may modulate migraine expression, especially in comorbid GI conditions.

### Limitations and future perspectives

This study is limited by its single-center design and may reflect only the patient population of an urban area. Furthermore, identifying a particular strain of *B. longum* with potential benefits for migraine warrants further exploration. Future large-scale, multicenter trials are needed to validate these results and explore the mechanisms linking microbial dysbiosis to migraine via immune, metabolic, and neurogenic pathways. At the preclinical level, we acknowledge that electrophysiological measures, such as trigeminocervical complex recordings, could provide additional insight into migraine-related neural activity. The absence of these assessments is a limitation of the present study and should be addressed in future experiments. Second, preclinical experiments in this study were performed only in male animals, whereas the clinical pilot trial included both sexes. Sex differences may influence pain, immune responses, and gut–brain interactions, potentially affecting the translational relevance of the findings. Future studies should incorporate both sexes in animal models to clarify these effects. In addition, we have not analyzed intestinal tissues collected from the animals to assess disease- and treatment-associated changes; this can be addressed in future studies.

## Conclusions

In this study, we identified distinct microbiome signatures in pediatric migraine patients with *B. longum* emerging as a key protective species, and different microbiome profiles in patients with or without GI disorders. The potential synergism of *B. longum* with *F. prausnitzii* and the association of *S. gallolyticus* with GI symptoms and disability underscore the complexity of migraine-microbiome interactions. These findings support future studies and the development of microbiota-targeted strategies, particularly *B. longum* supplementation, as promising adjunctive therapies for pediatric migraine management.

## Supplementary Material

Supplementary material supplement fig 2

Supplementary material S1 Figures

## Data Availability

The raw sequence data have been submitted to the GenBank Sequence Read Archive database under accession number PRJNA1111322.
