# Supplementary figures and images for: Targeting the microbiome in pediatric migraine: gastrointestinal manifestations and the therapeutic role of Bifidobacterium longum
Source: Gut Microbes. 2025 Dec 27;18(1):2606487. doi: 10.1080/19490976.2025.2606487 (PMC12758293; doi:10.1080/19490976.2025.2606487)

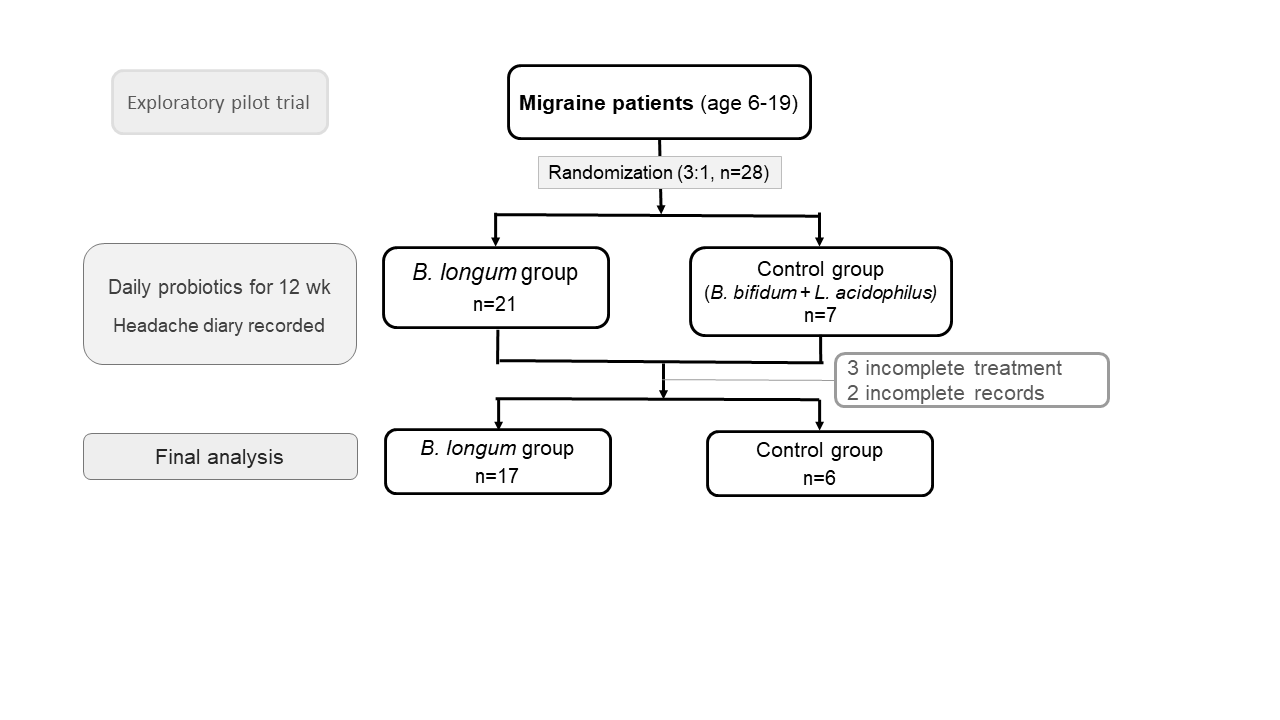

Supplement: Supplementary material — supplement fig 2 [file KGMI_A_2606487_SM7737.tif]
