# Supplementary material for: Targeting the microbiome in pediatric migraine: gastrointestinal manifestations and the therapeutic role of Bifidobacterium longum
Source: Gut Microbes. 2025 Dec 27;18(1):2606487. doi: 10.1080/19490976.2025.2606487 (PMC12758293; doi:10.1080/19490976.2025.2606487)
Supplement: Supplementary material — S1 Figures [file KGMI_A_2606487_SM7736.pdf]

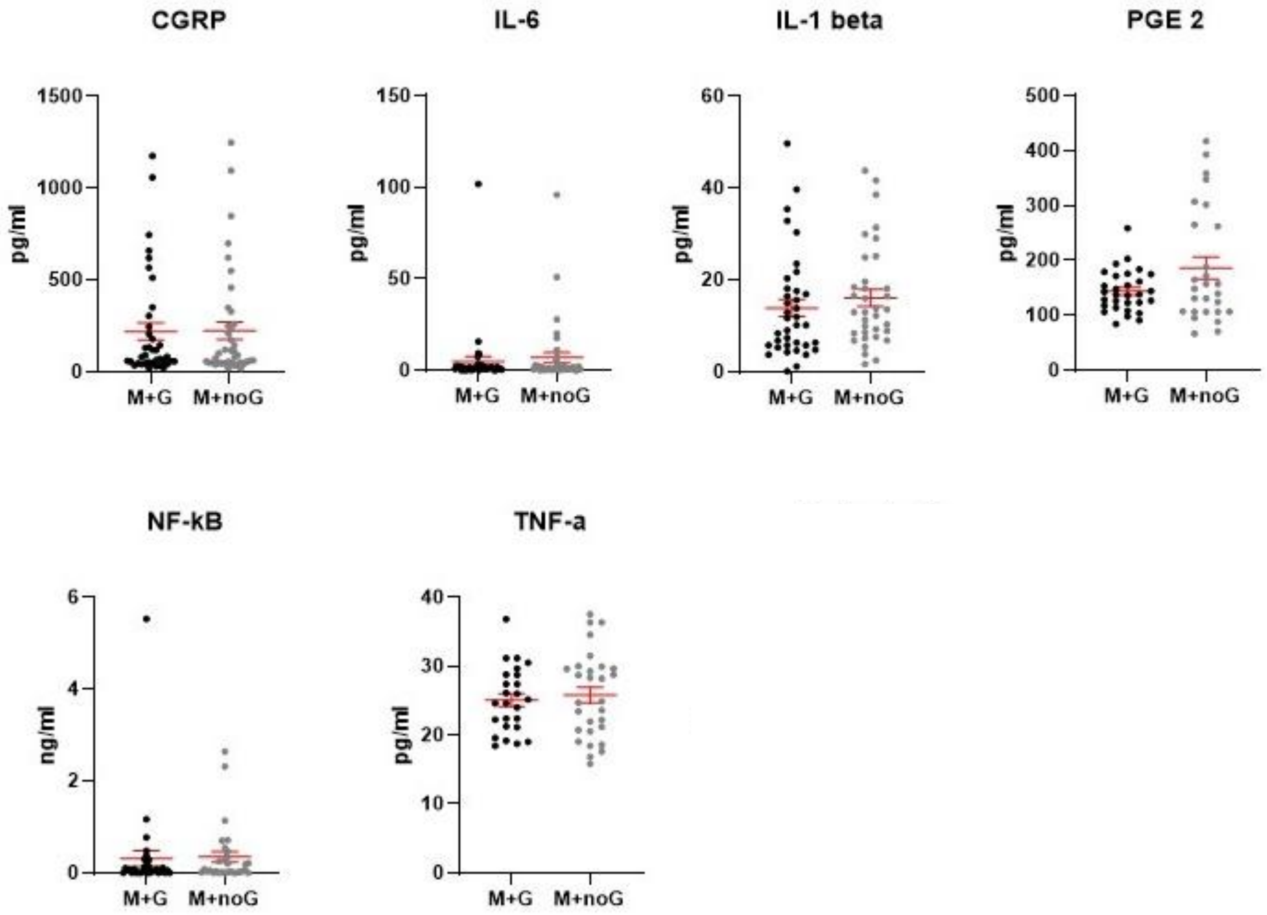

Additional Figure 1. Plasma CGRP and proinflammatory cytokines in migraineurs with (M+G) or without (M+noG) GI disorders. Plasma levels of CGRP and proinflammatory cytokines, including IL-6, IL-1 beta, PGE2, NF-kB, TNF-alpha, were detected with ELISA, and the datasets were evaluated by Student *t* test for two-group comparison.
